# Prevalence of tobacco smoking between 2009 and 2015 among students and the general population in the Kingdom of Saudi Arabia

**DOI:** 10.18332/tid/153975

**Published:** 2023-04-28

**Authors:** Omer Abid, Ali M. Alwadey, Kamal Eldeirawi

**Affiliations:** 1 Insight Research Institute, Flint, United States; 2 Tobacco Control Program, Ministry of Health, Riyadh, Kingdom of Saudi Arabia; 3 Department of Population Health Nursing Science, College of Nursing, University of Illinois Chicago, Chicago, United States

**Keywords:** cigarette smoking, systematic review, waterpipe smoking, shisha, Kingdom of Saudi Arabia

## Abstract

**INTRODUCTION:**

Tobacco smoking is a major risk factor for morbidity and mortality. Studies on smoking in the Kingdom of Saudi Arabia (KSA) have shown inconsistent results. The purpose of this study was to provide a literature review on the prevalence of tobacco smoking among school students, university students, and the general population of KSA during 2009–2015, before the implementation of new tobacco control measures.

**METHODS:**

We searched PubMed and Google for articles published in English from 2009 to 2015, focused on overall tobacco smoking and/or any form of tobacco smoking (e.g. tobacco, cigarette, and waterpipe) and conducted with a sample of ≥300 participants. Only the prevalence of current smoking was assessed. A narrative synthesis of the prevalence results was conducted.

**RESULTS:**

Of the 360 studies found in the primary search, 91 were selected for further examination for eligibility, and 20 studies were included in the review. Among school students, the prevalence of smoking any form of tobacco (cigarettes, waterpipes, or both) ranged 10.0–21.7%. The prevalence of cigarette smoking ranged 8.9–19.5% and for waterpipe smoking it was 9.5%. Among university students, the prevalence of smoking any form of tobacco ranged 11.1–25.3%, cigarette smoking 7.8–17.5%, and waterpipe 4.3–18.0%. In the general population, the prevalence of cigarette smoking ranged 19.6–23.5% and for waterpipe smoking it was 4.3%.

**CONCLUSIONS:**

Our study shows smoking levels were high in the KSA between 2009 and 2015. Studies utilizing standardized methodology with nationally representative samples are needed to better characterize the prevalence of different types of tobacco smoking. More research on national representative samples is needed, including studies on the same populations/groups/areas over time using standardized tools and definitions.

## INTRODUCTION

The worldwide prevalence of tobacco smoking is high. In 2013, the World Health Organization (WHO) estimated that 21% of adults (36% of men and 7% of women) globally were current smokers (i.e. smoking at the time of the survey, including daily and non-daily smoking of any form of tobacco, excluding smokeless tobacco)^1^. According to WHO, smoking prevalence is the highest in high-income countries, and 25% of adults were reported to be current smokers in 2013^1^. In contrast, 21% of adults living in middle-income countries and 16% in low-income countries were reported to be current smokers^1^. In 2013, WHO also estimated that the prevalence of current smoking in the Kingdom of Saudi Arabia (KSA) was 14%^1^.

Although preventable, tobacco consumption is a major modifiable risk factor for morbidity and mortality. In 2015, 6.4 million deaths were attributed to smoking worldwide^2^. Men accounted for >75% of these deaths and smoking was the second leading risk factor for attributable mortality among both genders in 2015^2^. Smoking is not considered socially acceptable, especially for women in the KSA, for cultural and religious reasons^3^. However, studies have shown that prevalence rates are considerably high for smoking among women in this country.

A previous systematic review was conducted to assess the prevalence of smoking in KSA among school students, university students, adults, and other population groups using studies published between 1987 and 2008^4^. The authors documented a wide range of smoking prevalence in KSA by reviewing 38 studies; however, most of the studies that included the study with the highest prevalence of 52.3% were only conducted on males and only performed in a certain region of KSA^4^. According to this review, this wide range was due to ‘different populations, using different criteria for current smoking, and estimating the prevalence in different regions, and at different times’^4^. Another review focused on studies that were published before October 2013 to determine the prevalence of smoking among middle/high school and college students in KSA^3^. Although results from this review revealed that there was a wide variation in the prevalence of smoking among the different studies, the review concluded that the prevalence of current smoking among students was alarmingly high. Additionally, a narrative review concluded that the prevalence of smokers almost doubled in KSA, especially in males, increasing from 21% in 1996 to 37% in 2012. Over the same period, tobacco smoking was estimated to cause over 280000 premature deaths in KSA^5^.

The Saudi Ministry of Health has a national tobacco control program that actively implements strategies and interventions to decrease tobacco smoking in the country. To better evaluate these efforts, more periodic reviews are needed to inform these strategies; however, the most recent review of the prevalence of smoking only included data up to 2008^4^. Therefore, this study reviewed the literature published during 2009–2015, in order to provide estimates of the prevalence of tobacco smoking in KSA among populations in that time period, including school students and university students, and before the implementation of more recent tobacco control efforts.

## METHODS

### Search strategy

A scoping review methodology was used. To identify original studies in English addressing the prevalence of tobacco smoking in KSA studies, we searched PubMed from January 2009 up to September 2015. The search strategy included the following terms: ‘smoking’, ‘tobacco’, ‘cigarette’, ‘waterpipe’, ‘shisha’, ‘prevalence’, ‘frequency’, and ‘Saudi Arabia’. Moreover, a supplementary search using Google was performed, using the same terms to capture additional articles that were not identified in PubMed. At a later stage, the reference lists of all articles selected (as well as review articles found) were screened to identify additional articles.

### Selection process

One reviewer originally screened the titles and abstracts of identified articles to determine potential eligibility; then, a full-text review (if retrievable) of those judged as potentially eligible was conducted. The same reviewer finally selected primary studies (not reviews) for the analysis that met the following criteria (based on full-text review): 1) the outcome assessed focused on overall tobacco smoking and/or any form of tobacco smoking (e.g. tobacco, cigarette, and waterpipe) by school students, university students, and/or the general population in KSA (this criterion was implemented to ensure that this review was comparable to the previous systematic review, which included articles up to 2008); 2) The participant recruitment strategy of each study involved a census of the population studied or a representative/random sample of the population studied; 3) the sample size comprised more than 300 participants; 4) studies were published between 2009 and 2015, and data were collected between 2007 and 2015; and 5) studies were published in English.

### Data extraction

The reviewer used a standardized form to extract data from the studies that were considered potentially eligible based on the abstract. This included the following data: author(s), year of publication; year(s) of data collection; place of sampling, including national or local coverage; sampling strategy (e.g. census, random sample, convenience sample); type of population (e.g. school students, university students, general population); sample size; response rate; age range/mean age; data collection instrument; definition of current smoking; overall current smoking prevalence (any form of tobacco, cigarette, and waterpipe); and gender differences if available. The study population was classified as the general population when subjects were randomly recruited from the registries of the primary healthcare centers.

Due to the large heterogeneity in the criteria to define current smoking, the data were classified as current smoking when it was described in the original reports as ‘current’, ‘in the past month/30 days’, ‘daily and occasional’, or ‘ever smoked and continues to smoke, even irregularly, but not a past smoker’. Since not all studies used the same terminology, we considered ‘daily and occasionally’ both as an indication of current smoking although the frequency is different.

### Data analysis

The studies were originally organized according to population type (i.e. school students, university students, and the general population); then according to the following forms of smoking: any tobacco smoking (cigarette, waterpipe, or both cigarettes and waterpipes), cigarette smoking, and waterpipe smoking; and finally according to national/local studies. Finally, the overall and by gender prevalence rates were described.

## RESULTS

**Figure 1:**
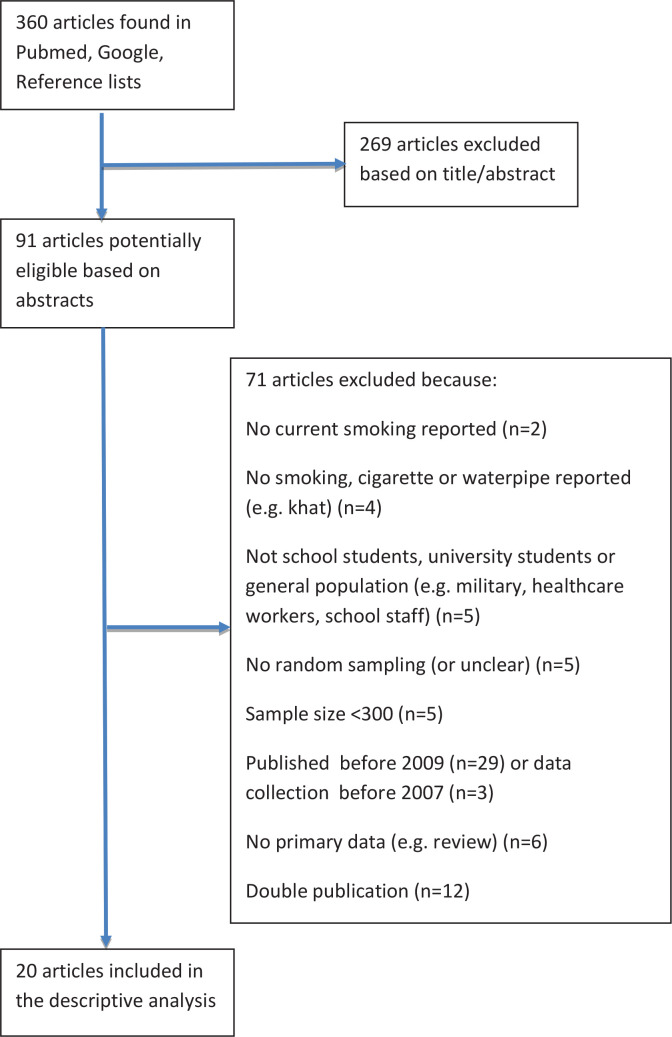
Flow chart of articles in the review process

In all, 360 articles were found in the primary search. After reviewing the titles and abstracts, 91 articles were selected, and the full-text articles were retrieved. A total of 53 articles that did not meet the above criteria were excluded (Figure 1). In addition, 6 articles were excluded because they summarized data from other studies, and 12 articles were excluded because they also described data that were published in other articles; however, these articles could be used to obtain information on the study characteristics. The remaining 20 studies were included in the descriptive analysis. We were unable to retrieve the full text for one of these studies; therefore, only the abstract was used for the analysis^6^.

### Students

One national study (the Global Youth Tobacco Survey) and 7 local studies that reported the prevalence of current smoking among school students in KSA were identified and met our criteria (Table 1). The local studies were conducted in Jeddah (n=2), Madinah Region (n=2), Riyadh (n=1), Al-Hassa (n=1), and Jazan (n=1)^7-14^. Although all studies focused on school students, the age of school students ranged from 11 to 22 years based on the type of school that was included in the study (i.e. intermediate and/or secondary school). Two studies included only male school students. The prevalence of current tobacco smoking (all forms) ranged from 10.0–21.7%. Additionally, the prevalence of current tobacco smoking (all forms) was higher among boys (16.2–37.0%) than among girls (3.8–9.1%). The prevalence of current cigarette smoking was higher (8.9–19.5%) than the prevalence of current waterpipe smoking (9.5%). The prevalence of cigarette smoking was also higher among boys (13.0–31.3%) than among girls (5.0–8/9%). Similarly, the prevalence of waterpipe smoking was higher among boys (13.3%) than among girls (6.1%). The study with the youngest respondents for cigarette smoking had the lowest prevalence rate (overall, boys and girls).

**Table 1:** Current smoking prevalence among school students in KSA (2009–2015)

*Study (Year)*	*Time of study*	*Location*	*Population*	*Age (years)*	*Sample size*	*Response rate (%)*	*Current smoking prevalence (%)*		
							*Overall*	*Male*	*Female*
**Any tobacco smoking** (all forms)**									
GYTS^7^ (2010)	2010	Country	Students, intermediate schools (grades 1–3)	13–15	2564	83.4	14.9*	21.2	9.1
Amin et al.^8^ (2012)		Al-Hassa	Students, secondary school	15–19	1652	84.6	21.7	30.3	8.5
Gaffar et al.^9^ (2013)	2011	Jazan	Students, intermediate and secondary school	13–21	3923	95.7	10.7	16.2	3.8
Fida et al.^10^ (2013)		Jeddah	Students, secondary school; males only	16–22	695	87.4		37.0	
Park et al.^11^ (2012)		Jeddah	Students, middle school (grades 7–9)	15–19	1019	85.9	10.0		
**Cigarette smoking**									
GYTS^7^ (2010)	2010	Country	Students, intermediate schools (grades 1–3)	13–15	2564	83.4	8.9	13.0	5.0
Al-Zalabani et al.^12^ (2015)	2013	Madinah region	Students, intermediate and secondary schools	11–19	3322	97.7	15.2	21.3	8.3
Al-Zalabani et al.^13^ (2015)	2014	Madinah region	Students, intermediate and secondary schools; males only		900	96.7		20.8	
Al Moamary et al.^14^ (2012)	2010	Riyadh	Students, high schools (grades 10–12)	15–18	1272	92.2	19.5	31.3	8.9
**Waterpipe smoking**									
GYTS^7^ (2010)	2010	Country	Students, intermediate schools (grades 1–3)	13–15	2564	83.4	9.5	13.3	6.1

* The current smoking prevalence may be lower. This estimate was based on data of ‘Current use of any tobacco product’, which includes 3.4% of participants who reported using smokeless tobacco. Some of the participants also smoke tobacco products, but the questions were not mutually exclusive.

One national study (the Global Health Professions Student Survey [GHPSS], 2010) and 7 local studies that reported the prevalence of current smoking among university students in KSA also met our search criteria (Table 2)^15-22^. Three of the local studies were conducted in Riyadh (among students of King Saud University), one study each was conducted in Al Hassa, Jazan, Jeddah, and Damman. Five of the eight studies focused on students in a health-related school (e.g. medicine, dental, nursing, etc.). The prevalence of current tobacco smoking (all forms) ranged 11.1– 25.3% and was considerably higher among males (14.7–32.7%) than among females (2.4–9.1%). The prevalence of current cigarette smoking was similar (7.8–17.5%) to the prevalence of current waterpipe smoking (4.3–19%). However, the prevalence of cigarette smoking was higher among males (10.8%) than among females (2%). Similarly, the prevalence of waterpipe smoking was higher among males (5.5–12.6%) than among females (2%).

**Table 2:** Current smoking prevalence among university students in KSA (2009–2015)

*Study (Year)*	*Time of study*	*Location*	*Population*	*Age (years)*	*Sample size*	*Response rate (%)*	*Current smoking prevalence (%)*		
							*Overall*	*Male*	*Female*
**Any tobacco smoking** (all forms)									
GHPSS^15^ (2010)	2010	Country	Students, 18 schools [dental, (applied) medical (sciences), nursing, pharmacy]		825	90.8	25.3		
Al Mohamed et al.^16^ (2010)	2006–2007	Al Hassa	Students, 9 colleges, King Faisal University; males only		1382	84.3–100		28.1	
Mahfouz et al.^17^ (2014)	2011–2012	Jazan	Undergraduate students in different higher education institutions	mean 20.9	3764	91.8	16.8	25.6	4.6
Wali et al.^18^ (2011)	2009–2010	Jeddah	Students, main Medical College King Abdulaziz University		643		14.0	24.8	9.1
Al Swuailem et al.^19^ (2014)		Riyadh	Dental students, King Saud University		400	67.0	17	27.6	2.4
Mandil et al.^20^ (2010)	2008–2009	Riyadh	Undergraduate students, King Saud University	17–25	6793	90.0	14.5	32.7	5.9
Subhan et al.^21^ (2009)	2006–2007	Riyadh	Students from King Saud University (Medicine, Dentistry, Pharmacy, Applied Medical Sciences), female nursing students from the National Guard Hospital	18–37	910	81.0	11.1	14.7	3.9
**Cigarette smoking**									
GHPSS^15^ (2010)	2010	Country	Students, 18 schools [dental, (applied) medical (sciences), nursing, pharmacy]		825	90.8	17.5		
Subhan et al.^21^ (2009)	2006–2007	Riyadh	Students from King Saud University (Medicine, Dentistry, Pharmacy, Applied Medical Sciences), female nursing students from the National Guard Hospital	18–37	910	81.0	7.8	10.8	2
**Waterpipe smoking**									
GHPSS^15^ (2010)	2010	Country	Students, 18 schools [dental, (applied) medical (sciences), nursing, pharmacy]		825	90.8	18		
Taha et al.^22^ (2010)	2008	Dammam	Male students of 3 colleges (medicine, applied medical sciences, dentistry), King Faisal University		371	74.2		12.6	
Subhan et al.^21^ (2009)	2006–2007	Riyadh	Students from King Saud University (Medicine, Dentistry, Pharmacy, Applied Medical Sciences), female nursing students from the National Guard Hospital	18–37	910	81.0	4.3	5.5	2

It must be noted that the GHPSS 2010 shows large differences in the prevalence of any tobacco smoking (all forms), cigarette smoking, and waterpipe smoking among the different student groups. The prevalence of any tobacco smoking (all forms) ranged from 5.9% among applied medical science students to 39.5% among dental students. The prevalence of cigarette smoking ranged from 2.9% among applied medical science students to 27.3% among pharmacy students, and the prevalence of waterpipe smoking ranged from 2.9% among applied medical science students to 34.3% among dental students.

### General population

Finally, two national studies and two local studies (1 conducted in the Eastern province and one in Jazan) met our criteria and reported the prevalence of current smoking in the general population in KSA (Table 3)^6,23-25^. Al-Turki et al.^6^ conducted a study in the Eastern province that included participants who were aged ≥30 years. Two other studies included participants who were aged ≥15 years, and the fourth study included participants who were aged ≥13 years. Moradi et al.^23^ and colleagues conducted a national survey study and revealed that the prevalence of current tobacco smoking (all forms) was 12.2% (21.5% among males and 1.1% among females)^23^. Al-Turki et al.^6^ conducted a large census study (n=196000) in the Eastern Province and determined that the prevalence of current tobacco smoking (all forms) was 16.9%. The prevalence of current cigarette smoking ranged 19.6–23.5% and was considerably higher among males (35.9%) than among females (2.3%)^24,25^.

**Table 3:** Current smoking prevalence among the general population in KSA (2009–2015)

*Study (Year)*	*Time of study*	*Location*	*Population*	*Age (years)*	*Sample size*	*Response rate (%)*	*Current smoking prevalence (%)*		
							*Overall*	*Male*	*Female*
**Any tobacco smoking** (all forms)									
Moradi-Lakeh et al.^23^ (2015)	2013	Country	Nationally representative sample	≥15	10735	89.4	12.2	21.5	1.1
Al-Turki et al.^6^ (2010)		Eastern province	Census	≥30	196268		16.9		
**Cigarette smoking**									
Albedah et al.^24^ (2011)	2008	21 cities	Sample	≥15	7003		19.6	35.9	2.3
Abdel Rahim et al.^25^ (2014)	2012	Jazan; 30 PHCs*	PHC attendees	≥13	4326	92.8	23.5		
**Waterpipe smoking**									
Moradi-Lakeh et al.^23^ (2015)	2013	Country	Nationally representative sample	≥15	10735	89.4	4.3	7.3	1.3

* Primary Healthcare Centers.

## DISCUSSION

We evaluated the results of peer-reviewed studies that focused on the prevalence of tobacco smoking in KSA among three population groups (school students, university students, and the general population) from 2009 to 2015, thus allowing us to assess the baseline for changes in prevalence that are expected from 2015, in light of the changes in tobacco control in the KSA.

The prevalence of smoking tobacco (all forms) in that time period was found to be high in all three population groups: school students (10.0–21.7%), university students (11.1–25.3%), and the general population (12.2–16.9%). The prevalence of cigarette smoking was higher in the general population (19.6–23.5%) than among school students (8.9–19.5%) and university students (7.8–17.5%). Finally, the prevalence of waterpipe smoking was slightly lower among school students (9.5%) than among university students (4.3–19.0%) and the general population (4.3%).

In all population groups, the prevalence of all forms of tobacco smoking was much higher among males than among females, and this gender difference was more pronounced among the general population compared to students (especially school students). The male–female difference could be because smoking is considered more taboo among females than it is among males^4^, and this may have resulted in decreased smoking among females. Additionally, this effect may be less pronounced among younger age groups. However, the general understanding is that females smoke more than they report because of the taboo or social desirability bias. The latter could also, but to a much lesser extent, hold among males. A systematic review found that reports of smoking are underestimated when smoking prevalence is based on self-report than when it is based on biological samples^26^.

Similar to earlier reviews^3,4^, we observed that there was a wide range in the prevalence of current smoking among the different studies included in the current review. These differences may be due to differences in the population groups included (e.g. age, socio-economic status, and study area), time of data collection, geographical locations, and methodological approaches (e.g. operational definition of smoking, sample size, sampling technique, and response rate). For example, since response rates vary (67–97.7%), they may have introduced selection bias, especially in studies with lower response rates. Moreover, studies used different operational definitions of current smoking. Furthermore, studies also varied in sample sizes and the targeted populations, and, therefore, in the precision of the prevalence estimates. Variability in the prevalence estimates necessitates more standardized studies, such as the Global Adult Tobacco Survey (GATS), with nationally representative samples.

Based on our systemic review and previous reviews, limited national studies have been performed in the KSA. In the previous systematic review identified, there was only one study which was a community study. Jarallah et al.^27^ conducted this study, between 1990 and 1993 with a large multi-stage sample of participants who were aged ≥15 years, and revealed that the overall prevalence was 11.6%, which was very similar to the 12.2% prevalence observed in the national study that was conducted by Moradi-Lakeh et al.^23^ in 2013 that was included in our review also using a multi-stage nationally representative study.

Although this study did not intend to survey the current literature, it is striking that a more current study conducted by Muzammil et al.^28^ on university students between 2016 and 2017 documented that the prevalence of waterpipe smoking was 32.2%. It is also likely that the prevalence of waterpipe smoking is higher now with no end seen in its increasing prevalence going forward without interventions^29^.

### Strengths and limitations

This study had some strengths. First, this study allows us to assess tobacco prevalence in KSA which includes both students and the general population within the 2009–2015 study period. Second, we used a comprehensive search strategy and included methodological quality criteria in our selection process. However, there are some limitations that should be considered. First, the search, selection of studies, and data extraction were all conducted by one reviewer; ideally, this should have been conducted by two independent reviewers (and disagreements should have been resolved through consensus with a third reviewer)^30^. However, this was not possible because of resource constraints. Furthermore, we included studies in the review with varying definitions of current smoking, which possibly contributed to the wide range in reported prevalence rates (together with differences in the age range and the time of data collection). However, other reviews encountered this problem^31,32^. Further, no meta-analysis was performed. Instead, a narrative synthesis of the results was conducted due to the differences between the studies and limitations in the reported data.

## CONCLUSIONS

The review found that the most relevant studies were performed on a certain population subgroup, such as school and university students, in a specific area of the country. There is limited evidence for the whole country (as was also found in a systematic review on waterpipe tobacco smoking^33^) and for the adult population. Therefore, more research on national representative samples is needed, including studies on the same populations/groups/areas over time using standardized tools and definitions. In particular, the variability in the range of estimates warrants studies that are conducted using the GATS, which was carried out in more than 25 low- and middle income countries, to provide more accurate and precise prevalence estimates as well as information on associated factors. Furthermore, research on the implementation and effectiveness of tobacco control policies across the country should be conducted. Given the high rates of smoking in these studies, and the exponential increase of waterpipe in the country and elsewhere^29^, strategies aimed at smoking prevention and tobacco control are needed. Most importantly, there is a need to continually increase the retail price of tobacco products through higher taxes because it is the single most effective way to decrease consumption and encourage tobacco users to quit according to the WHO and tobacco control experts^34^. This is especially important given the high burden of non-communicable diseases in the country^35,36^, with smoking being an important risk factor. Further studies are warranted that are conducted using the top methodological standards of the Global Adult Tobacco Survey (GATS) designed by the World Health Organization. Fortunately, KSA conducted the GATS survey in 2019 with these far more precise and accurate results now available at the following link^37^. Any future studies should use the GATS methodology.

## Data Availability

The data supporting this research are available from the authors on reasonable request.
